# Eravacycline for *Mycobacterium abscessus* infections: pharmacodynamic advantages of long-acting post-antibiotic effects and weekly dosing regimens

**DOI:** 10.1128/spectrum.01473-25

**Published:** 2026-03-30

**Authors:** Ruoyan Ying, Guoling Yang, Xiaochen Huang, Jie Wang, Li Wang, Wei Sha

**Affiliations:** 1 Shanghai Key Laboratory of Tuberculosis, Shanghai Pulmonary Hospital, Tongji University School of Medicine 481875, Shanghai, People's Republic of China; 2 Tuberculosis Center for Diagnosis and Treatment, Shanghai Pulmonary Hospital, Tongji University School of Medicine 481875, Shanghai, People's Republic of China; University of Nebraska Medical Center, Omaha, Nebraska, USA

**Keywords:** *Mycobacterium abscessus*, eravacycline, PAE, PA-SME, pulmonary infection

## Abstract

*Mycobacterium abscessus* (MAB) is one of the most common non-tuberculous mycobacteria with an extremely high resistance rate to classic anti-tuberculosis drugs. This study aimed to evaluate the activities of the novel tetracycline agent eravacycline (ERC) against MAB using clinical isolates and mouse infection models. We determined the minimum inhibitory concentration (MIC) of ERC against the MAB standard strain ATCC 19977 and 27 clinical isolates. The THP-1-derived macrophage infection model was established to assess the intracellular inhibitory activity. The post-antibiotic effect (PAE) and post-antibiotic sub-MIC effect (PA-SME) of ERC were evaluated against ATCC 19977. Additionally, the efficacy and safety of treatment of ERC under different dosing intervals were compared with omadacycline (OMC) and tigecycline (TGC) in a BALB/c mouse model. The MIC_50_ and MIC_90_ of ERC were 0.03 and 0.5 μg/mL, respectively. In macrophages, 1 μg/mL ERC inhibited bacterial growth by 21.65% after 24 h, outperforming both 5 μg/mL TGC (4.85%) and OMC (17.41%). ERC also exhibited a prolonged PAE (up to 10 days after 2 h exposure to 16 μg/mL) and PA-SME (>7 days, at 0.5 μg/mL), longer than those of TGC and OMC. In the intracellular model, ERC and OMC showed sustained antibacterial activity for up to 1 week after drug removal, whereas TGC did not. In mice, ERC treatment reduced lung bacterial burden by 3.34–3.74 log_10_CFU after 2 weeks. Notably, a once-weekly high-dose regimen of ERC achieved therapeutic efficacy comparable to that of alternate-day dosing and significantly reduced inflammatory lung injury. ERC demonstrates potent *in vitro* and intracellular activity against MAB, supported by an exceptionally long PAE/PA-SME and promising *in vivo* efficacy and safety. The sustained antibacterial effect supports the feasibility of a weekly dosing regimen, positioning ERC as an ideal candidate for the treatment of MAB infections.

**IMPORTANCE:**

*Mycobacterium abscessus* (MAB) infections are increasingly prevalent and notoriously difficult to treat due to intrinsic resistance to most antibiotics. This study demonstrates that eravacycline—a novel tetracycline—exhibits exceptional potency against MAB, outperforming current therapies like tigecycline and omadacycline. Key findings include eravacycline’s low minimum inhibitory concentration (MIC) values (MIC_50_ = 0.03 µg/mL), strong intracellular activity, and uniquely prolonged post-antibiotic effects. Crucially, a weekly high-dose regimen in mice achieved bacterial clearance comparable to daily dosing while reducing lung inflammation, leveraging eravacycline’s sustained pharmacological impact. This challenges the need for frequent dosing, potentially minimizing toxicity and improving patient adherence.

## INTRODUCTION

*Mycobacterium abscessus* (MAB) is one of the most clinically prevalent non-tuberculous mycobacteria (NTMs) (1, 2) and is the etiological agent of severe pulmonary infections in vulnerable patients. Epidemiologic evidence suggests that there has been an overall increase in MAB infections and diseases over the past decade (3). The prevalence of pulmonary NTM disease has increased from 1.3 to 7.9 cases per 100,000 population in Asia, with MAB being one of the most prevalent strains (4, 5). Although China is one of the 30 high-burden countries of tuberculosis, the increasing prevalence of NTM isolates among all mycobacterial-positive strains is also of concern. A national survey showed that 6.4% of mycobacterial isolates were NTM instead of MTBC, with MAB accounting for the proportion at 35.96% (6).

Because MAB is naturally resistant to most anti-TB drugs, only a limited number of antimicrobial agents are effective in treating MAB infections. Treatment regimens recommended by ATS guidelines include multiple antimicrobial agents, containing a macrolide, amikacin (AMK), a β-lactam, and another repurposed antibiotic (7–9), such as linezolid, clofazimine, and tigecycline (TGC) (10–13). Even with the use of these antibiotic combinations, the treatment outcome remains poor, with the average cure rate around 50% (14, 15), as well as the inconvenience of injectable agents’ usage and high rate of adverse effects.

Tetracycline is a kind of old, broad-spectrum antibiotic that may inhibit bacterial protein synthesis. Previous studies have demonstrated that TGC shows a potent inhibitory effect against MAB (16, 17). However, TGC is frequently responsible for serious adverse events, such as gastrointestinal discomfort, like uncontrollable nausea and vomiting. Therefore, there is an urgent need for effective antibiotics with few adverse effects for the treatment of MAB diseases. Preclinical studies have shown that another tetracycline, omadacycline (OMC), has antimycobacterial activity against MAB in mouse models and *in vitro* experiments (18, 19). An observational clinical study with a small sample also found that OMC could improve the treatment outcome of MAB pulmonary disease (20). These results demonstrate that tetracyclines may become one of the core antibiotics in the regimen of MAB treatment in the future.

Eravacycline (ERC) is a newly developed fully synthetic tetracycline derivative with potent broad-spectrum activity against a wide range of microorganisms (21). Whether it could be a stronger anti-MAB drug than TGC and OMC, we conducted a study to explore the activities of ERC against MAB infections *in vitro*, *ex vivo*, and *in vivo* and to optimize the administration dose as well.

## MATERIALS AND METHODS

### Bacterial strains

Twenty-seven clinical isolates of MAB obtained from NTM-infected patients in Shanghai Pulmonary Hospital between January 2022 and December 2023 were included in this study. The isolates from patients suspected of having tuberculosis were tested by the proportion method using the BACTEC MGIT 960 system (Becton Dickinson, Franklin Lake, NJ, USA). The strains were preliminarily classified as NTM using a *p*-nitrobenzoic acid-containing medium and then identified at the species level by sequencing (Zeesan Biotech Co., Ltd., Xiamen, Fujian, China).

These isolates were stored in 7H9 broth (Becton Dickinson, Franklin Lakes, NJ, USA) containing 15% glycerol in a freezer at −80°C until analysis. The test isolates were grown at 37°C in Middlebrook 7H9 broth (Becton Dickinson, Franklin Lake, NJ, USA) supplemented with 10% ADC (5% bovine serum albumin, 2% dextrose, and 5% catalase).

### Antimicrobial agents

ERC was gained from Everest Medicines Company (Shanghai, China). OMC was gained from Tonglian Group (Shenyang, Liaoning, China). TGC, doxycycline (DOX), minocycline (MIN), AMK, and clarithromycin (CLA) were purchased from Med Chem Express (New Jersey, USA). All dry powders were prepared by dissolving in DMSO to produce a solution. All drug solutions were filtered and sterilized with a 0.22 µm sterile filter and stored separately in a freezer at −80°C.

### Minimum inhibitory concentration assay

The broth microdilution minimum inhibitory concentration (MIC) assay of ERC was performed as described previously (22). Following the recommendations of the Clinical and Laboratory Standards Institute and with some modifications, a series of double dilutions of the testing antimicrobial agents were prepared for each isolate using the following drug concentration ranges: 0.03–16 mg/L for ERC and OMC, 0.015–4 mg/L for TGC, 0.12–16 mg/L for DOX, 1–8 mg/L for MIN, 1–64 mg/L for AMK, and 0.06–64 mg/L for CLA (23).

To prepare drug plates, 20 µL of drug-containing coating buffer was added to sterile 96-well plates using an automatic dispenser, followed by air-drying, sealing, and storing at 4°C. To determine MIC, the test isolates were grown to mid-log phase (OD_590_ ≈ 0.4, ~2.5 × 10^8^ CFU/mL) and diluted with broth medium to a final concentration of ~10^5^ CFU/mL for the testing.

A total of 200 µL of bacterial suspension was added to each plate. After 3–5 days of incubation at 37°C, the positive control wells were observed, and a positive result was defined by visible bacterial precipitation at the bottom of the well. All drug results were read at 99% inhibition. Reference strains of *S. aureus* (ATCC29213) and *E. coli* (ATCC25922) were used to perform the test system. The MIC test was performed in triplicate to ensure the reliability of the results.

### Time-kill and growth curve for ERC

The activities of the ERC alone against the MAB standard strain ATCC 19977 were determined. The strain was grown in 7H9 broth to the exponential phase, and a suspension at an optical density OD_590_ ≈ 0.4 was prepared by diluting the culture in fresh broth. Culture tubes (5 mL) containing ERC at 0.5, 1, 2, 4, 8, and 16 μg/mL specific to each MAB standard strain ATCC 19977 in 4.8 mL 7H9 broth were prepared and inoculated with 200 μL of the MAB standard strain ATCC 19977 suspension. A positive control for growth of the MAB standard strain ATCC 19977 without ERC was included in each assessment. The samples were incubated in an orbital shaker at 220 rpm and 37°C. At 0, 1, 3, and 7 days, a 50 μL aliquot was obtained from each sample, and appropriate 10-fold serial dilutions were prepared in 7H9 broth, inoculated onto 7H11 agar, and CFU enumerated after 3–5 days of incubation at 37°C. Similarly, to determine the growth curve of ERC in MAB standard strain ATCC 19977, cultures diluted in broth medium to a final concentration of ~10^5^ CFU/mL were exposed to different concentrations of ERC (0.125–16 μg/mL) and incubated in an orbital shaker at 220 rpm and 37°C. OD_600_ was measured every 24 h intervals.

### Intracellular inhibitory effect

First, we established a macrophage model, starting from human myeloid leukemia mononuclear cells (THP-1), which were differentiated into macrophages using 100 ng/mL phorbol 12-myristate 13-acetate (24, 25). The bacterial solution was turbid to 1 mg/mL (1 × 10^7^ CFU/mL) and diluted to a multiplicity of infection (MOI) of 5. After 3 h of infection, the supernatant was removed and washed three times with phosphate-buffered saline (PBS) to remove the bacteria that had not entered the macrophages. Medium containing 10, 5, and 1 μg/mL ERC was used in the experimental group, medium containing 5 μg/mL OMC/TGC was used as a positive control (26), and medium without added drugs was used as a negative control (NC).

After 24 h, the bacteria were washed three times with PBS, and then incubated with 1% Triton X-100 for 10 min. The digested bacteria were then subjected to three 10-fold dilutions (10^−1^ to 10^−3^), followed by inoculation of 50 μL of the bacterial solution onto 7H11 agar plates. The plates were incubated at 37°C in 5% CO_2_ for 3 or 5 days, and then the CFU on each plate was enumerated.

### Post-antibiotic effect assay

To select the optimal dosing regimen, we subsequently investigated the post-antibiotic effect (PAE) and post-antibiotic sub-MIC effect (PA-SME) of ERC. PAEs of ERC and control drug were analyzed using a previously described methodology (27, 28). Log-phase MAB standard strain ATCC 19977 was exposed to different concentrations of ERC (0.5–16 μg/mL), TGC at a concentration of 2 μg/mL, and OMC at a concentration of 4 μg/mL for 2 h in 7H9 broth (29). The same concentrations of bacteria with drug-free 7H9 broth were used as a positive control, while media without bacteria or drugs were used as a negative control. After centrifuging and washing the bacteria three times to remove the antibiotic, the washed bacterial cells were resuspended in 7H9 broth and incubated at 37°C until they reached growth saturation (OD_max_). The OD_600_ of each culture was determined before drug exposure, after drug removal, and at 24-hour intervals thereafter. The PAE duration was calculated as the time taken for the antibiotic-treated culture to reach 50% OD_max_ of the drug-free culture minus the time taken for the drug-free control to achieve the same point.

### PA-SME assay

Log-phase MAB standard strain ATCC 19977 was exposed to ERC at a concentration of 16 μg/mL for 1 week and incubated in an orbital shaker at 220 rpm and 37°C. After centrifugation to remove the drug, bacteria were resuspended and adjusted to 0.5 McFarland standards and diluted 1:1,000 in antibiotic-free broth or broth containing 0.1, 0.25, or 0.5 μg/mL of ERC for continuous measurement of OD_600_. PA-SME was defined as *T*_pa_ − *C*. *T*_pa_ is the time required for cultures previously exposed to 16 μg/mL of ERC and then exposed to different sub-MICs to grow and reach 50% OD_max_ in drug-free cultures, and *C* is the corresponding time for the growth control that was not exposed to ERC. The sub-MIC effect (SME) was induced in the same way as the PA-SME, without the prior induction of PAE. The SME was defined as *T*_*s*_ − *C*. *T*_*s*_ is the time required for the cultures exposed only to sub-MICs to grow and reach 50% OD_max_ of the drug-free culture.

### Effects of the PAE on intracellular inhibitory activity

To evaluate the PAE of antibiotics within macrophages, THP-1-derived macrophages were infected with MAB ATCC 19977 at a low MOI (0.1). This lower bacterial load was chosen to establish a sustainable infection model over the extended duration (1 week) required for effects of the PAE on intracellular antibacterial activity observation, preventing excessive bacterial overgrowth and macrophage lysis in the control groups. Macrophages infected with MAB ATCC 19977 were treated with different concentrations of ERC, 5 μg/mL OMC, or 5 μg/mL TGC, and incubated for 24 h. After washing out the drug-containing medium, incubation was continued for 1 week, following operations as defined in Materials and Methods (Intracellular inhibitory effect).

### Mouse infections and treatment

The immunocompetent BALB/c female mice (five mice per group per timepoint, aged 6–8 weeks old) were acquired from Tongji University and transferred to an Animal Biosafety Level 2 (ABSL-2) laboratory (LENSCI Biotechnology Co., Ltd., Kunshan, Jiangsu, China), reaching a weight of 20 g before infection. BALB/c mice were infected with MAB standard strain ATCC 19977 (via a nasal drop) at 2.0 × 10^6^ CFU/20 µL (30, 31).

The background groups were sacrificed the day after infection to determine the CFUs in the lungs at the start of treatment. The other six groups were injected intraperitoneally as follows: negative control: 200 µL of PBS (the DMSO diluted in PBS as a control solution) per mouse; ERC: 200 µL per mouse (15 mg/kg); ERC: 200 µL per mouse (60 mg/kg); OMC: 200 µL per mouse (15 mg/kg); OMC: 200 µL per mouse (60 mg/kg); TGC: 200 µL per mouse (15 mg/kg). The adult dose of ERC in humans is 100 mg per day, which is equivalent to 15 mg/kg for mice (32, 33).

We established two dosing regimens. The 15 mg/kg dose group was injected intraperitoneally on alternate days, while the 60 mg/kg dose group was injected intraperitoneally once a week. The cumulative doses in these two groups were the same. Once the dosing interval is prolonged, the blood concentration of a drug drops below the MIC for a period of time, so there is a need to rely on the PAE to maintain therapeutic efficacy. Combined with PK/PD data for ERC from Andes et al., in the administration range of 2.5–80 mg/kg, the elimination half-life (*t*_1/2_) is 3.9–17.6 h (34), while the time to elimination of 98% of the drug in the body after a single dose is 23.4–105.6 h (~1–5 days). For the group with more frequent administration and given the intraperitoneal mode of administration, we administered the drug every other day to reduce stress and injury to the mice.

The left lung tissue was taken for pathological sections, and the right lung tissue was homogenized in 1 mL of PBS. The lung homogenates were serially diluted (10^−2^, 10^−3^, and 10^−4^), and 50 µL of the contents of the dilution tubes was spread on 7H11 agar containing ampicillin (10 µg/mL), polymyxin B sulfate (200 U/mL), amphotericin B (10 µg/mL), and trimethoprim lactate (10 µg/mL). These plates were incubated at 37°C in 5% CO_2_ for 5 days, after which the CFU of ATCC 19977 on each plate was enumerated.

### Histopathological analysis

After the mice were sacrificed, the lung tissue was isolated. Left lung tissue was immediately fixed in 4% paraformaldehyde for 24 h, dehydrated with wax, and embedded in paraffin. The embedded sections were cut into 5 μm slices for staining. Dewaxed and rehydrated sections were heated in citric acid buffer at 121°C for 30 min to restore antigenic activity. Acid-fast bacteria staining solution was used to observe the mycobacteria, while hematoxylin and eosin (H&E) staining was used to observe the histopathology. The pathology was evaluated by pathologists in a blinded manner.

### Statistical analyses

All experimental data were analyzed using GraphPad Prism 8 software (San Diego, CA, USA). CFU data from *in vitro* studies were analyzed to determine the mean ± standard error of the mean (SEM) for each concentration or timepoint in each experimental group. Comparisons of numerical data were performed using one-way ANOVA. *P* values of less than 0.05 (*P < 0.05; **P < 0.01; ***P < 0.005; ****P < 0.001) were considered statistically significant.

## RESULTS

### MICs of ERC and comparator antimicrobials against MAB clinical isolates and standard strain

MAB standard strain ATCC 19977 and 27 clinical isolates were collected for antibiotic susceptibility testing. MIC_50_ and MIC_90_ of ERC were 0.03 and 0.5 μg/mL, respectively. The results for the tested tetracyclines are summarized in Table 1. The MIC values for ERC against the standard strain and most clinical isolates were generally one- to threefold lower than those for OMC*,* and only eight clinical isolates had the same MIC for ERC and OMC. The results demonstrated that ERC had comparable antimicrobial activity *in vitro* against MAB standard strains and clinical isolates.

**TABLE 1:** Distribution of MIC values for eravacycline and comparator drugs against 27 MAB clinical isolates and standard strain ATCC 19977

		MIC (μg/mL)						
Strain	Subspecies	ERC	OMC	TGC	DOX	MIN	AMK	CLA
KL1ZCC	*M. abscessus subsp. abscessus*	0.125	0.25	1	>16	>8	8	4
KL2ZWL	*M. abscessus subsp. abscessus*	0.125	0.25	1	>16	>8	8	0.5
KL5LWJ	*M. abscessus subsp. abscessus*	0.06	0.25	0.5	>16	>8	16	>16
KL6HJ	*M. abscessus subsp. abscessus*	0.06	0.25	1	2	>8	16	≤0.06
KL8HFZ	*M. abscessus subsp. abscessus*	0.125	1	0.25	>16	>8	8	>16
KL9CHJ	*M. abscessus subsp. abscessus*	0.03	0.03	0.03	8	>8	2	0.25
KL10TXF	*M. abscessus subsp. abscessus*	0.03	0.03	1	>16	>8	16	>16
KL11GFJ	*M. abscessus subsp. abscessus*	0.03	0.06	0.03	>16	>8	2	8
KL12MYF	*M. abscessus subsp. massiliense*	0.03	0.03	0.25	>16	>8	4	1
KL13WXH	*M. abscessus subsp. abscessus*	0.03	0.03	0.5	>16	>8	8	16
KL14CS	*M. abscessus subsp. abscessus*	0.03	0.03	0.5	>16	>8	4	>16
KL15CYF	*M. abscessus subsp. abscessus*	0.5	1	0.25	16	8	8	1
KL16CSL	*M. abscessus subsp. abscessus*	0.03	0.125	1	>16	>8	8	>16
KL17CCY	*M. abscessus subsp. abscessus*	0.03	0.25	0.5	16	>8	8	2
KL18HKP	*M. abscessus subsp. abscessus*	0.03	0.125	1	>16	>8	16	>16
KL19LJ	*M. abscessus subsp. bolletii*	0.06	0.25	2	>16	>8	16	0.25
KL20LJ	*M. abscessus subsp. abscessus*	0.03	0.25	0.5	>16	>8	16	>16
CLA-C1SLF	*M. abscessus subsp. abscessus*	0.03	0.03	0.06	8	>8	2	0.125
CLA-C2WQY	*M. abscessus subsp. abscessus*	0.25	0.5	4	>16	>8	32	0.25
CLA-C4HMD	*M. abscessus subsp. abscessus*	0.125	0.5	0.5	>16	>8	32	>16
CLA-C5LJY	*M. abscessus subsp. abscessus*	0.125	0.5	1	>16	>8	32	0.5
CLA-C6SYN	*M. abscessus subsp. abscessus*	>16	>16	>4	>16	>8	>64	>16
CLA-C8WYM	*M. abscessus subsp. abscessus*	0.125	0.25	0.25	>16	>8	32	2
CLA-C9CQ	*M. abscessus subsp. abscessus*	0.5	1	1	>16	>8	16	>16
CLA-C10HGY	*M. abscessus subsp. abscessus*	0.03	0.06	1	>16	8	16	>16
CLA-S1ZMH	*M. abscessus subsp. massiliense*	0.125	1	2	>16	>8	32	16
KLS1YXN	*M. abscessus subsp. massiliense*	0.03	0.03	4	>16	>8	8	>16
ATCC 19977		0.03	0.25	0.03	2	>8	2	0.125
	MIC_50_	0.03	0.25	0.5	8	8	8	1
	MIC_90_	0.125	1	2	>16	>8	32	>16

According to the CLSI breakpoints for susceptibility for MAB, the values for these antimicrobial agents were 2 μg/mL for CLA, 16 μg/mL for AMK, and 4 μg/mL for TGC (35). Among the 27 clinical isolates, only 1 isolate (CLA-C6SYN) was resistant to all tested drugs, 4 were TGC-resistant (including 2 intermediate isolates, MIC = 2 µg/mL), and 22 were TGC susceptible. The ERC MICs of the two TGC-resistant isolates (CLA-C2WQY and KLS1YXN) were 0.03 and 0.25 μg/mL, respectively. In addition, DOX and MIN were relatively ineffective against MAB clinical isolates than other tetracyclines.

### Antimicrobial activity of the ERC with different concentrations

To generate insight into the activity of ERC over time, we performed time-kill assays in combination with growth curve analysis using the standard strain ATCC 19977.

**Fig 1:**
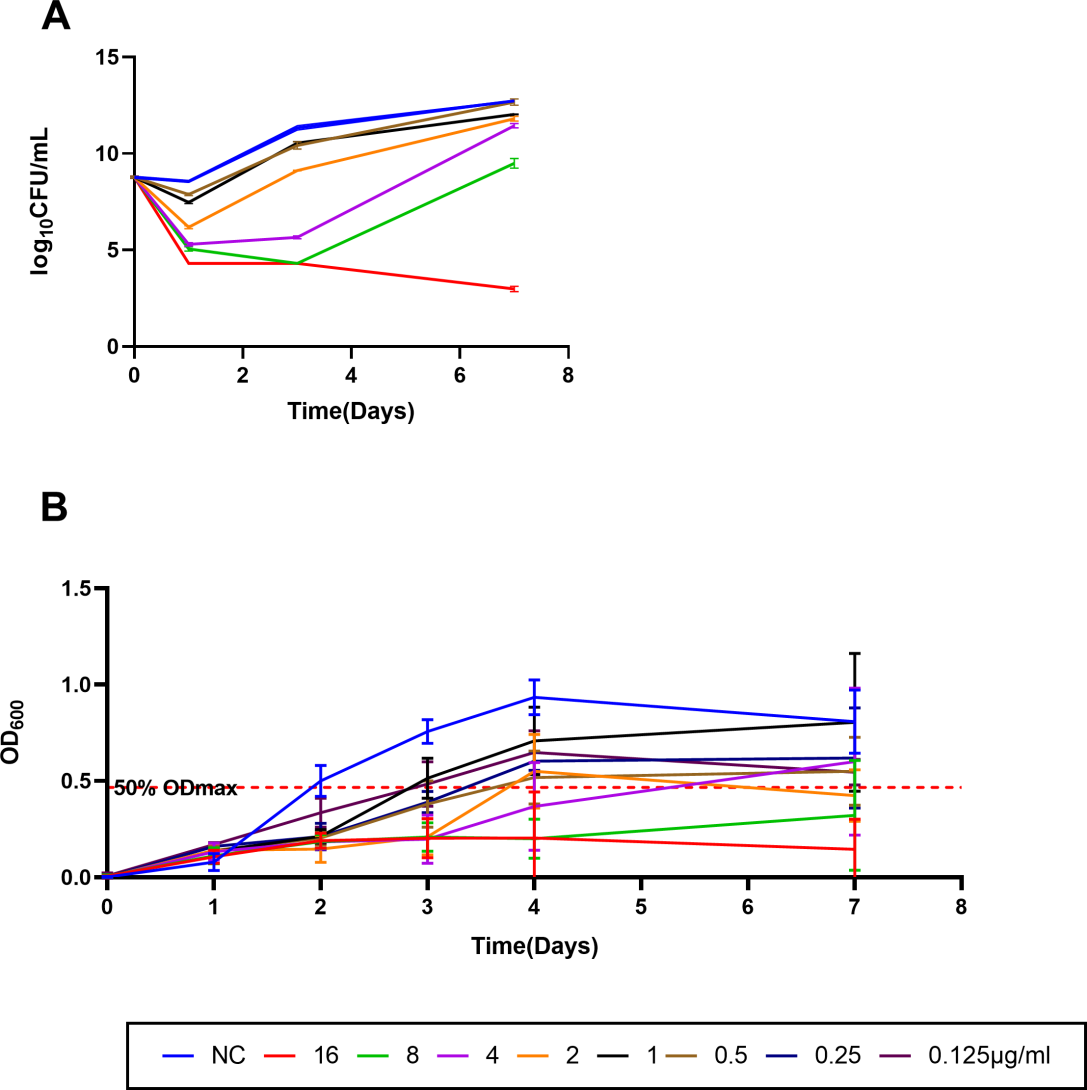
Time-kill activity of ERC against (**A**) MAB ATCC 19977 was exposed to ERC at 0.5, 1, 2, 4, 8, and 16 μg/mL to each isolate and to no drug in 7H9 broth. No drug was used as a negative control. Surviving colonies were recovered on 7H11 agar in duplicate at 1, 3, and 7 days and enumerated. Growth curves of (**B**) MAB ATCC 19977 in the presence of the different concentrations (0.125–16 μg/mL) of ERC. No drug was used as a negative control. The red dotted line represents 50% OD_max_ in drug-free cultures. All data are shown as mean ± SEM (*n* = 6).

Time-kill experiments demonstrated that ERC showed concentration-dependent antimicrobial activity, as demonstrated during the first 24 h of exposure, where ERC at 0.5 μg/mL and increasing fold concentrations above the 0.5 μg/mL reduced the CFU levels. Further reductions in CFU occurred until 3 days when the bacteria were exposed to 8 μg/mL or higher concentrations of ERC. At the highest concentration tested (16 μg/mL), the CFU level continued to decline through day 7, but no elimination was achieved. However, at lower concentrations, bacterial regrowth occurred after 3 days of exposure (Fig. 1A).

Complementing the time-kill results, growth curve monitoring by OD_600_ measurements provided consistent insights into the bacteriostatic and bactericidal dynamics of ERC. At lower concentrations, ATCC 19977 resumed growth after 3–4 days, reflecting the reversible inhibition observed in the CFU counts. However, at higher concentrations (8–16 μg/mL), the OD_600_ values remained persistently low throughout the 7-day observation period, confirming sustained bacterial suppression without recovery. These findings demonstrate that increasing ERC concentrations not only enhance the degree of inhibition but also prolong the duration of antibacterial activity (Fig. 1B). These results underscore the potent and persistent antimicrobial effect of ERC against MAB, supporting its potential utility in prolonged or intermittent dosing regimens.

### Intracellular inhibitory effect

**Fig 2:**
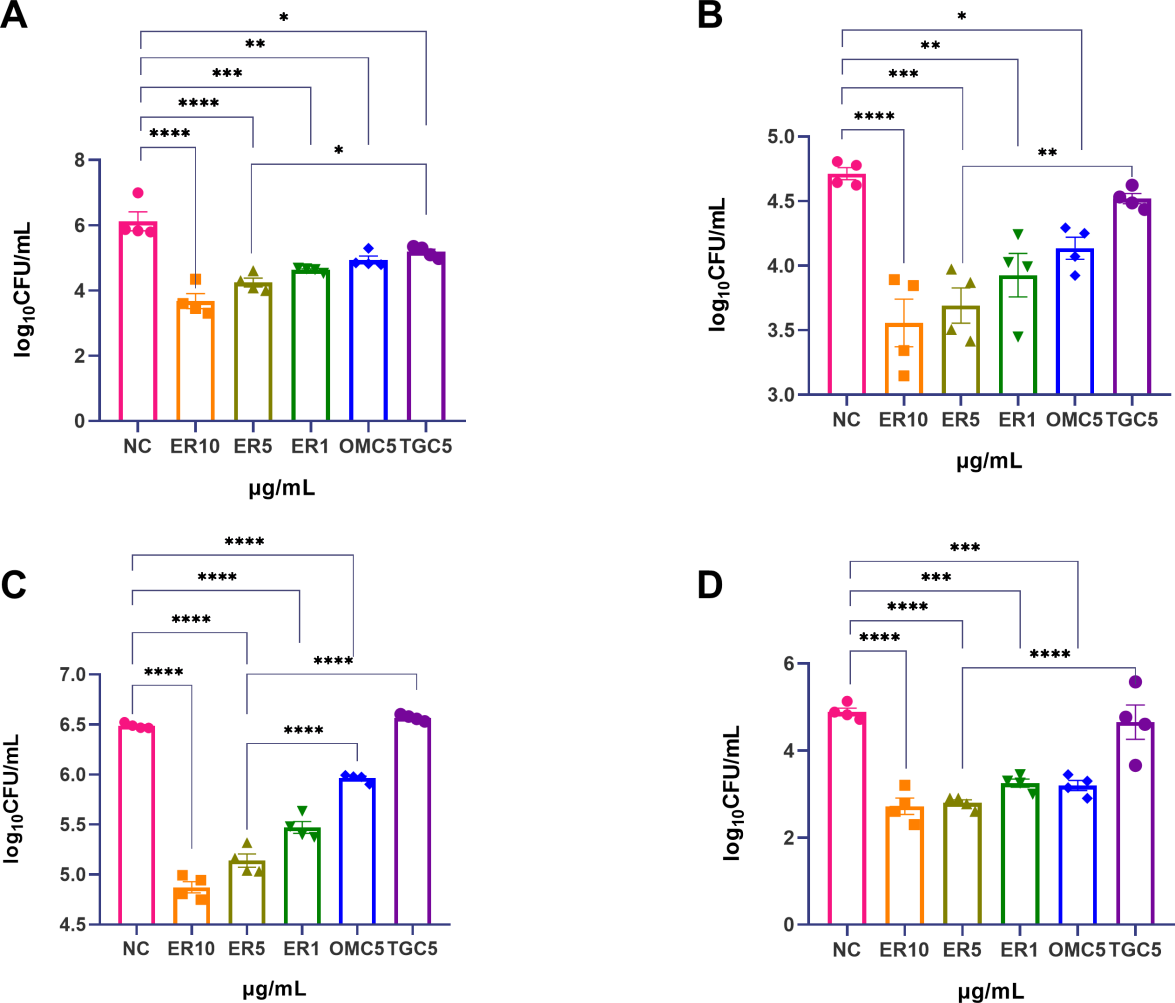
Intracellular inhibitory activity of different concentrations of ERC, as well as OMC and TGC (positive control), against ATCC 19977 in macrophages at an MOI of 5. (**A**) MAB ATCC 19977. (**B**) Clinical isolate, KL10TXF. (**C**) Clinical isolate, KLS1YXN. (**D**) Clinical isolate, KL8HFZ. All data are shown as mean ± SEM (*n* = 4). *P < 0.05; **P < 0.01; ***P < 0.005; ****P < 0.001.

In the macrophage MAB infection model, we further evaluated the efficacy of ERC against four independent MAB strains (ATCC 19977 and recent pulmonary clinical isolates KL10TXF, KLS1YXN, and KL8HFZ). The three recent clinical isolates have a range of MIC values for the antibiotics most frequently used to treat MAB infection (Table 1), AMK (8–16 μg/mL), CLA (>16 µg/mL), and TGC (0.25–4 mg/mL). As summarized in Fig. 2, ERC exhibited significantly stronger growth inhibition than the NC group across all tested strains (*P* < 0.01). The percent inhibition of ERC at 1, 5, and 10 μg/mL on intracellular was as follows: 20.77%, 29.21%, and 35.70% for ATCC 19977; 16.69%, 21.69%, and 24.54% for the clinical isolate KL10TXF; 15.65%, 20.76%, and 24.87% for the clinical isolate KLS1YXN; 33.51%, 42.80%, and 44.34% for the clinical isolate KL8HFZ. These results indicated that the growth inhibition of ERC on intracellular bacterial growth was concentration dependent.

We compared the intracellular activity of ERC with OMC and TGC at an equal concentration of 5 μg/mL, a level established for meaningful comparison of anti-mycobacterial efficacy in macrophages (26). In Fig. 2 and Tables S1, 5 μg/mL ERC exhibited significantly stronger inhibition than both OMC and TGC across all tested strains (*P* < 0.05). Specifically, the percent inhibition of ERC was 29.21%, 21.69%, 20.76%, and 42.80% for ATCC 19977, KL10TXF, KLS1YXN, and KL8HFZ, respectively. In contrast, OMC showed percent inhibition of 17.41%, 12.27%, 8.05%, and 34.56%, while TGC showed only 4.85%, 4.12%, −1.26%, and 4.80%, respectively. These results clearly demonstrate the superior intracellular activity of ERC over OMC and TGC at the same concentration.

### PAE of ERC, OMC, and TGC against the MAB standard strain

To select the optimal dosing regimen, we further investigated the PAE of ERC (36, 37). Following 2 h of pulse exposure to different concentrations (0.5, 1, 2, 4, 8, and 16 µg/mL) of ERC, 4 µg/mL OMC, and 2 µg/mL TGC, the growth of ATCC 19977 was monitored. The PAE was calculated as defined in Materials and Methods (PAE assay).

**Fig 3:**
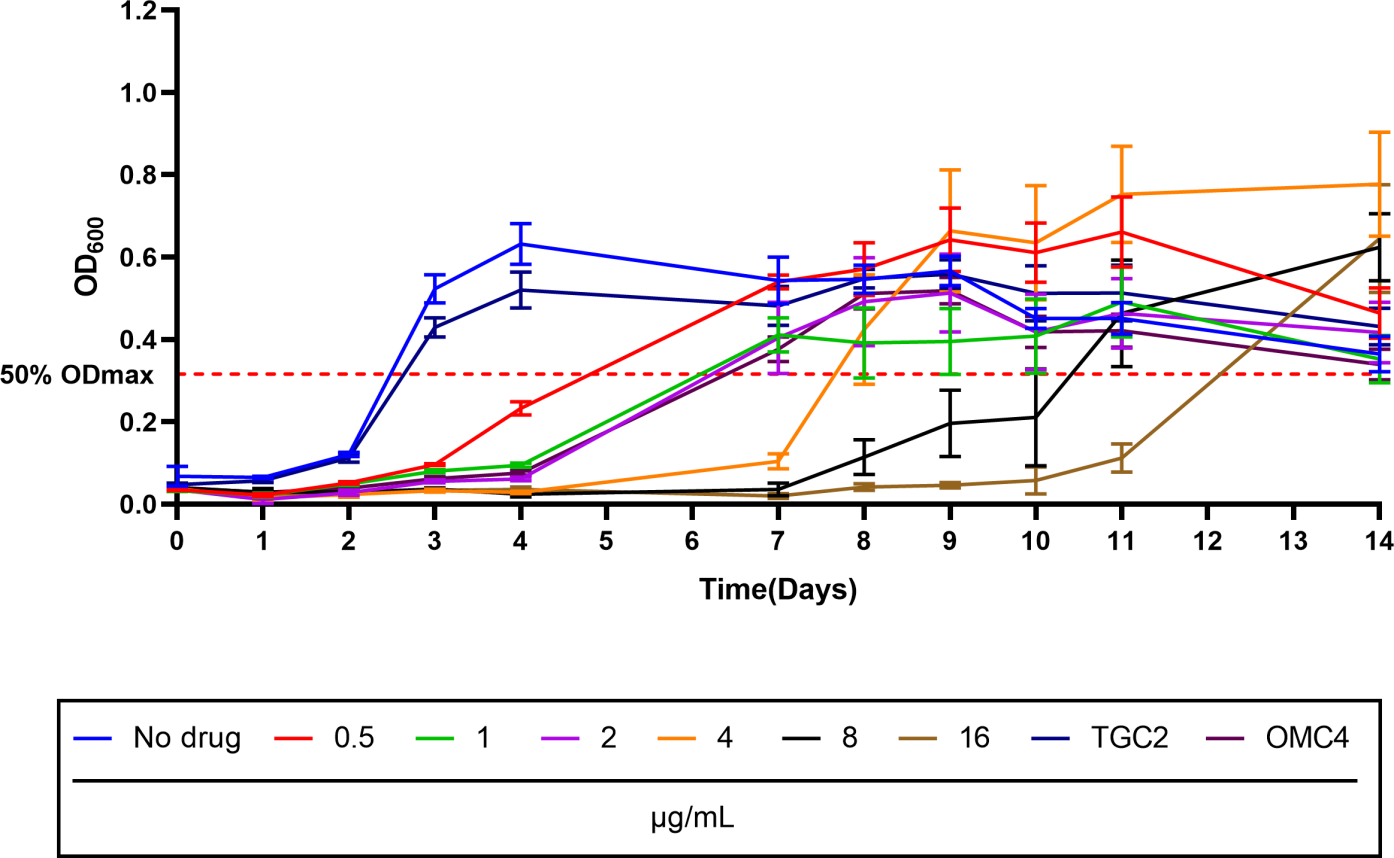
PAE durations of ATCC 19977 after pulsed dosing with different concentrations of ERC. OMC and TGC were used as positive controls, and no drug was used as a negative control. The red dotted line represents 50% OD_max_ in drug-free cultures. All data are shown as mean ± SEM (*n* = 6).

As quantified in Fig. 3 and Table 2, the PAE of ERC was concentration dependent: 10 (12.5–2.2) days for 16 µg/mL, 8 (10.3–2.2) days for 8 µg/mL, 5 (7.7–2.2) days for 4 µg/mL, 3 days (5.7–2.2; 6–2.2) for both 1 and 2 µg/mL, and 2 (4.6–2.2) days for 0.5 µg/mL (the number of days rounded to an integer). These PAE values are days longer than those observed in the drug-free control group. The PAE for 4 µg/mL OMC was 4 (6.9–2.2) days, similar to that of 1 and 2 µg/mL ERC. In contrast, the PAE for 2 µg/mL TGC was extremely short (<0.5 days) and comparable to that of the untreated control. These results clearly demonstrate that ERC induces longer PAE compared to OMC and TGC, with the effect lasting up to 10 days at the highest concentration tested (16 µg/mL).

**TABLE 2:** Time to reach 50% OD_max_ in post-antibiotic effect (PAE) assays for MAB standard strain ATCC 19977

	μg/mL								
	No drug	ERC0.5	ERC1	ERC2	ERC4	ERC8	ERC16	TGC2	OMC4
Time to 50% OD_max_ (days)	2.2	4.6	5.7	6	7.7	10.3	12.5	2.6	6.9

### PA-SME of ERC against MAB standard strain

We next evaluated the PA-SME of ERC. MAB standard strain ATCC 19977 was exposed to ERC at 16 µg/mL for 1 week. After drug removal, the bacteria were resuspended in broth containing sub-MIC concentrations of ERC (0.1, 0.25, or 0.5 µg/mL). The PA-SME was calculated as *T*_pa_ − *C*, as defined in Materials and Methods (PA-SME assay).

**Fig 4:**
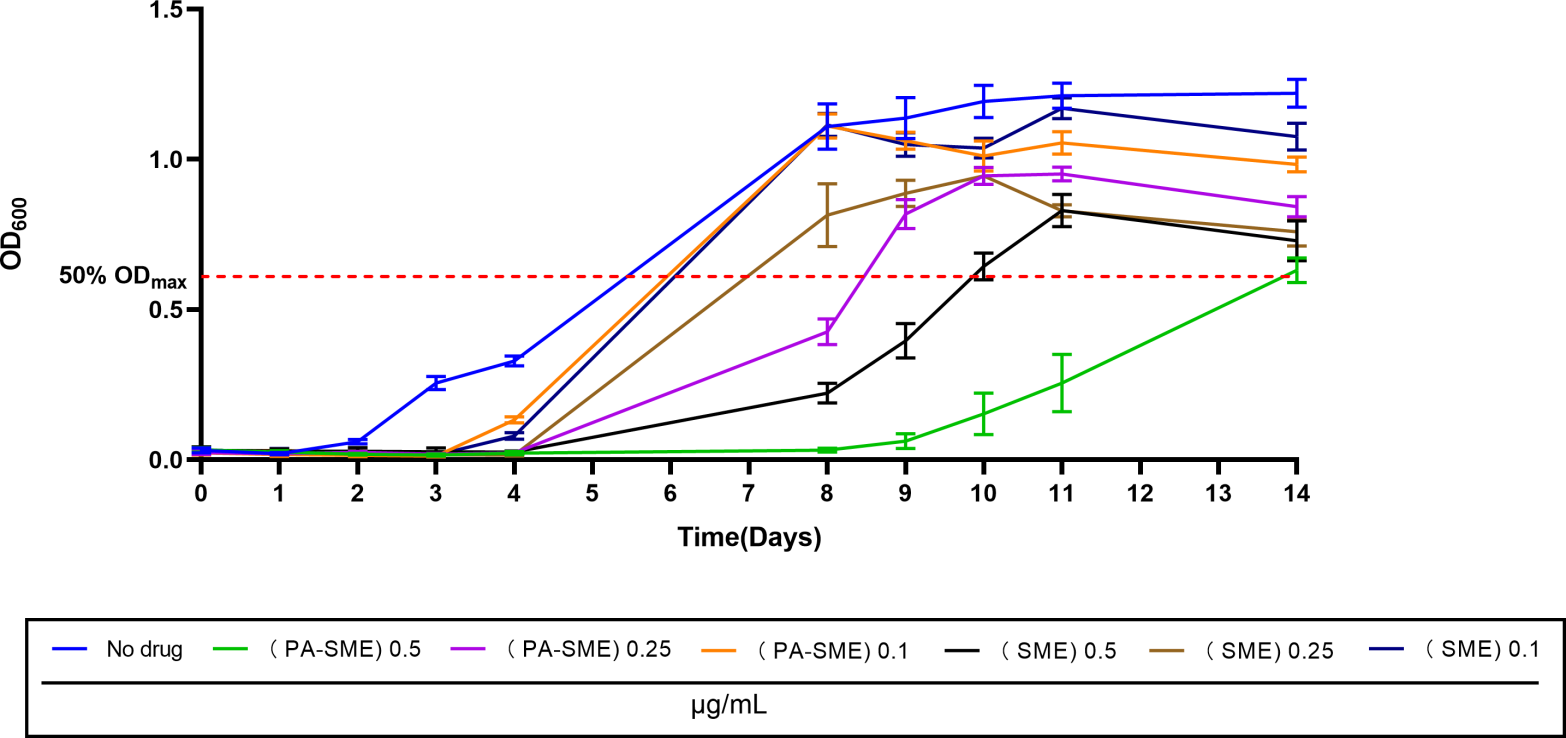
PA-SME durations after initial exposure to 16-fold MIC of ERC for 1 week. The green, purple, and orange lines are the growth curves of ATCC 19977 after 1 week of exposure to ERC of 16 μg/mL. The black, brown, and navy lines are the growth curves of ATCC 19977 incubated in drug-free Middlebrook 7H9 broth for 1 week. The red dotted line represents 50% OD_max_ in drug-free cultures. All data are shown as mean ± SEM (*n* = 6).

As shown in Fig. 4 and Table 3, the PA-SME increased with the sub-MIC concentration of ERC: 8 (13.8–5.6) days for 0.5 µg/mL, 2 (8–5.6) days for 0.25 µg/mL, and <1 day for 0.1 µg/mL. For comparison, the SME measured without prior exposure to a high concentration of ERC was shorter: 4 (10.3–5.6) days for 0.5 µg/mL MIC, 2 (7.3–5.2) days for 0.25 µg/mL, and <1 day for 0.1 µg/mL (the number of days rounded to an integer). This demonstrates that ERC exposure induces an increased sensitivity of ATCC 19977, resulting in prolonged suppression of bacterial growth even at sub-inhibitory concentrations during the post-antibiotic phase.

**TABLE 3:** Time to reach 50% OD_max_ in post-antibiotic sub-MIC effect (PA-SME) and sub-MIC effect (SME) assays for MAB standard strain ATCC 19977

	μg /mL						
	*C*	*T*_pa_			*T*_*s*_		
	No drug	PA-SME 0.5	PA-SME 0.25	PA-SME 0.1	SME 0.5	SME 0.25	SME 0.1
Time to 50% OD_max_ (days)	5.6	13.8	8	6.4	10.3	7.3	6.5

### Sustained intracellular antibacterial activity of ERC, OMC, and TGC after drug removal

To assess the durability of the antibacterial effect within macrophages after antibiotic exposure, we designed an experiment to compare bacterial regrowth following drug removal. THP-1-derived macrophages infected with MAB ATCC 19977 (MOI = 0.1) were treated for 24 h with different concentrations of ERC (1, 5, and 10 μg/mL), or with 5 μg/mL of OMC or TGC. Following this initial treatment, the drug-containing medium was removed, the cells were washed, and they were then incubated in drug-free medium for an additional week (designated as week 1, or W1). As controls, parallel groups of infected macrophages were either continuously exposed to the same antibiotics for the entire 1-week period or left untreated.

**Fig 5:**
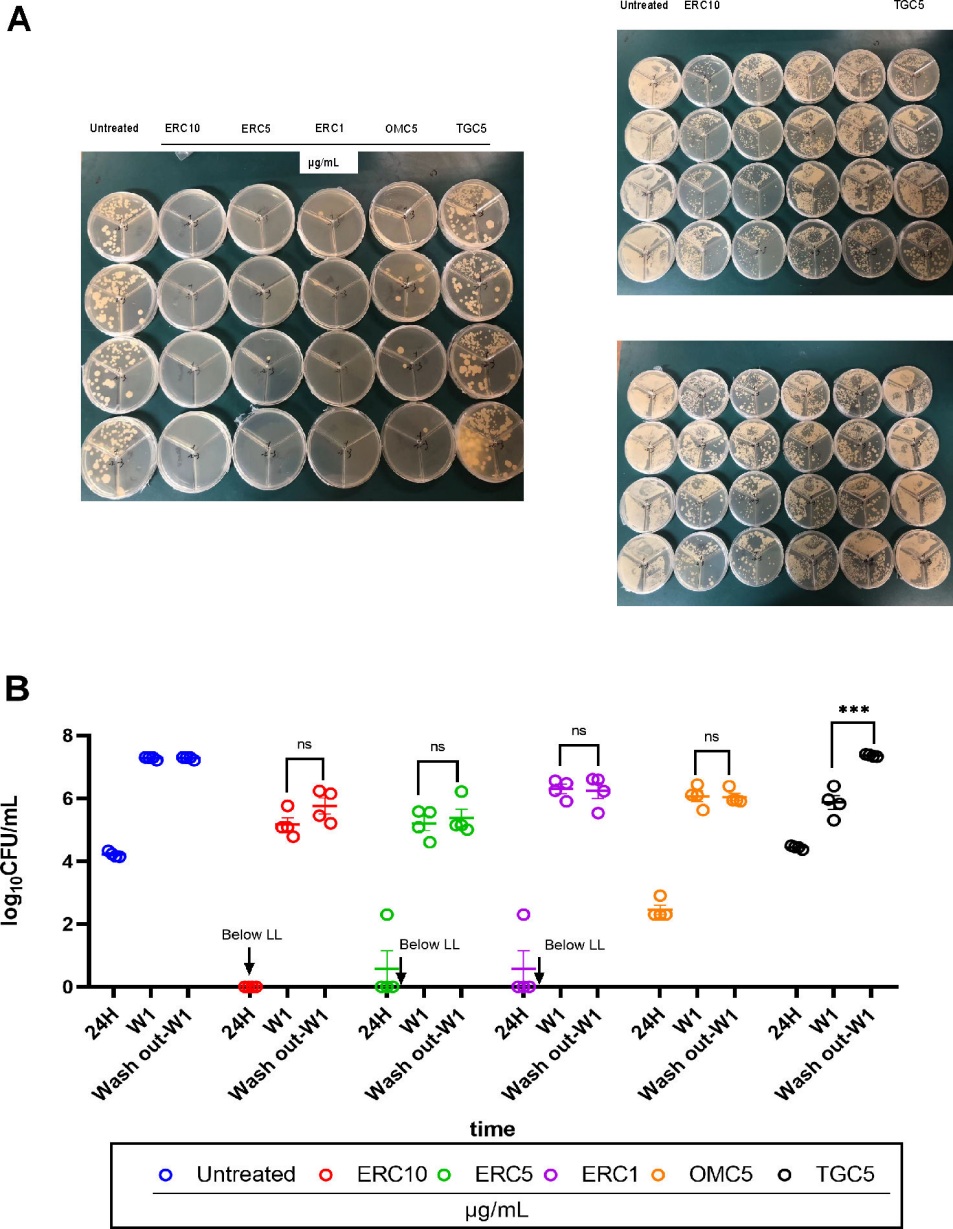
Sustained intracellular antibacterial activity of ERC, OMC, and TGC after drug removal. (**A**) Schematic diagram of the experimental timeline. The left panel shows the intracellular inhibitory effects of each group of drugs after 24 h. The upper right panel presents the intracellular inhibitory effects under the continuous administration of each group of drugs for 1 week. The lower right panel presents the effect of sustained intracellular antibacterial activity after drug removal. (**B**) Quantitation of bacterial CFUs in macrophages infected with ATCC 19977; LL, lower limit of quantification; the arrow represents below LL (mean ± SEM of *n* = 4). ***P < 0.005; ns, not significant.

As quantified in Fig. 5B, both ERC and OMC exhibited a prolonged intracellular antibacterial effect that persisted for up to 1 week after drug removal. The bacterial counts (CFU) in the “drug removal” groups at W1 remained significantly lower than those in the untreated control group and were comparable to the counts in the corresponding drug-containing medium groups. This indicates that a brief 24 h pulse of ERC or OMC is sufficient to suppress intracellular bacterial growth for an extended period.

In contrast, the inhibitory effect of TGC was short lived. One week after the removal of TGC (5 μg/mL), bacterial counts rebounded to levels similar to the untreated control and were significantly higher (*P* < 0.005) than in macrophages continuously exposed to TGC. This demonstrates a lack of sustained PAE for TGC in the intracellular environment.

Furthermore, at the same initial concentration of 5 μg/mL, ERC demonstrated a stronger sustained inhibitory effect than OMC 1 week after drug removal, as evidenced by lower residual CFU counts.

### Efficacy of ERC against MAB ATCC 19977 pulmonary infection in mice model

In the untreated group, mice receiving PBS treatment at the same frequency as the test group, the lung burden of ATCC 19977 fell by approximately 1.5 log_10_ CFU over the first week and then fell slowly in the second week. Without immunosuppressive intervention, the strains of BALB/c mice infected in the untreated group were not auto-cleared by the experimental endpoint (Fig. S2). Meanwhile, in the positive control group, which received OMC or TGC treatment, ATCC 19977 lung burden for all regimes fell by approximately 3 log_10_ CFU reduction over 2 weeks.

**Fig 6:**
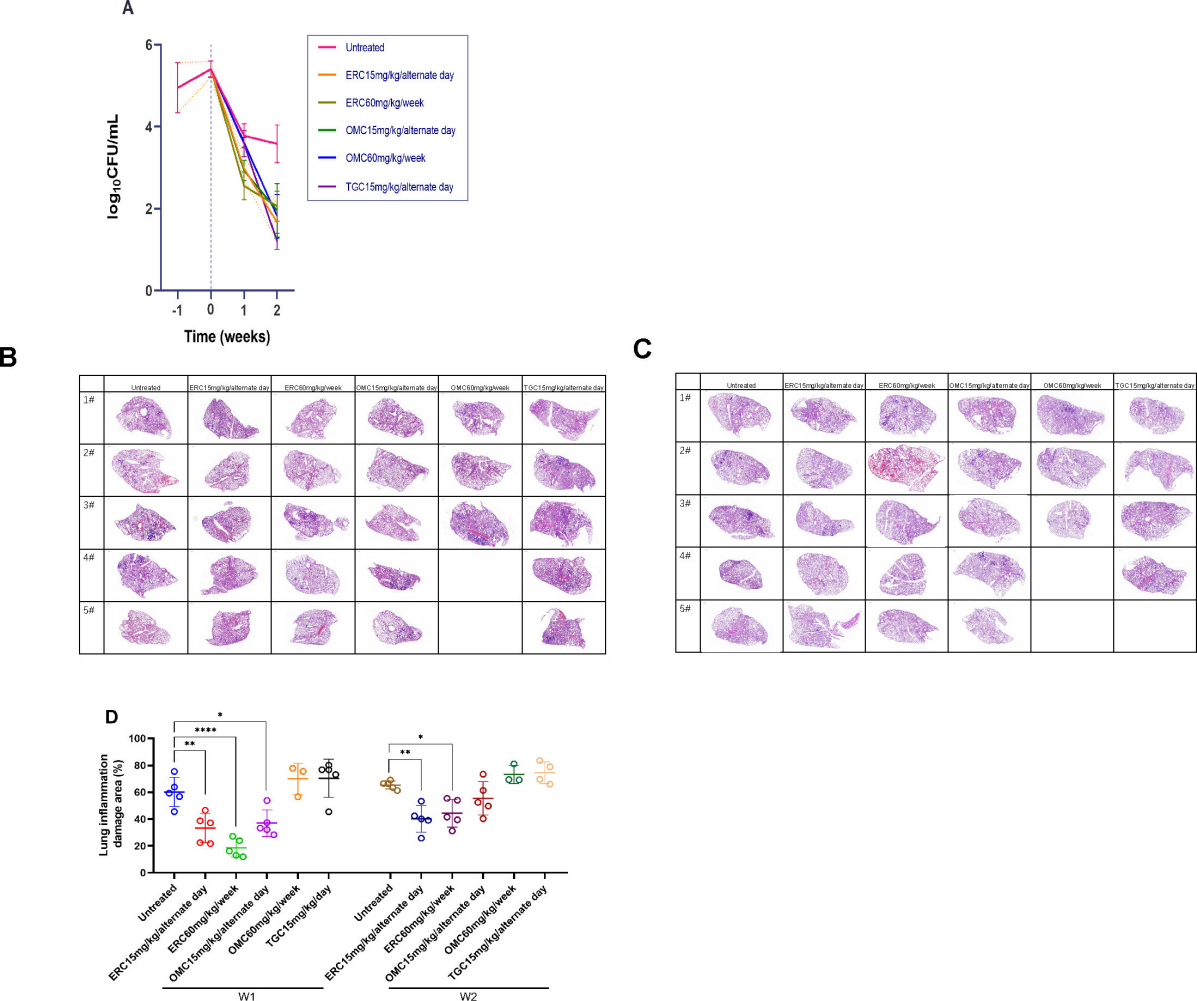
Efficacy of ERC against MAB ATCC 19977 pulmonary infection in mice model. (**A**) Bacterial CFUs of samples from the lungs of mice infected with ATCC 19977 (mean ± SEM, *n* = 5 per group per time point). Week −1 represents the day after mice were infected with ATCC 19977, and week 0 represents the day of antibiotic treatment initiation (denoted by a vertical dotted line). The untreated group was administered with PBS on alternate days. The alternate-day dose of OMC, ERC, and TGC was 15 mg/kg. The weekly dose of OMC and ERC was 60 mg/kg. All agents were administered by intraperitoneal injection. (**B**) Histopathology of lung sections (the largest surface of the left lung was taken) from infected mice after 1 week of drug treatment. (**C**) Histopathology of lung sections (the largest surface of the left lung was taken) from infected mice after 2 weeks of drug treatment. BALB/c mice were infected with ATCC 19977 by nasal drop (~2.0 × 10^6^ CFUs) for 1 week and intraperitoneally injected with ERC (1.5 mg/mL, 200 μL on alternate days)*,* ERC (6 mg/mL, 200 μL once a week), OMC (1.5 mg/mL, 200 μL on alternate days)*,* OMC (6 mg/mL, 200 μL once a week)*,* or TGC (1.5 mg/mL, 200 μL on alternate days) for another 2 weeks. #1–5 indicate representative lung sections from five mice, and the absence of pictures represents the animal’s death before the endpoint. The quantitation of inflammatory areas is shown in each section. (**D**) Quantitation of areas of inflammatory damage (the area of inflammatory damage was delineated, and the whole section of the lung lobe was compared using CaseViewer software; see Fig. S3 and Table S3 for more details) in the lungs of mice infected with MAB ATCC 19977 (mean ± SEM, *n* = 5). *P < 0.05; **P < 0.01; ****P < 0.001.

The treated group CFU counts were approximately 2 log_10_ lower than the untreated group at week 2. After 2 weeks of treatment, ERC alternate-day dosing, ERC weekly dosing, OMC alternate-day dosing, OMC weekly dosing, and TGC alternate-day dosing resulted in an average reduction in lung burden of 3.74, 3.34, 3.44, 3.58, and 4.20 log_10_CFU, it is referring to the CFU count at time 0, respectively (Fig. 6A; Table S2).

During the first week of treatment, with each treatment regimen, ATCC 19977 was reduced faster than in untreated mice. In particular, the decrease was most pronounced with ERC weekly dosing, which reduced the number of bacteria in the lungs of mice by 2.84 log_10_CFU. The lung burden reductions with alternate-day dosing of ERC and OMC were comparable at 2.42 and 2.47 log_10_CFU. Lung burdens with OMC weekly dosing and TGC alternate-day dosing were reduced by an average of 1.78 and 1.82 log_10_CFU.

In the second week, all treatment regimens reduced ATCC 19977 lung burden and thus exhibited bactericidal activity (Fig. S1). However, both the OMC weekly dosing group and the TGC treatment group had mice die before the end of the study, with mortality rates of 40% and 10%. The OMC weekly dosing regimen showed a high mortality rate in the first week of the experiment. Whole lung H&E staining of the dead mice revealed extensive congestion and hemorrhage in the lungs, with blood pooling in the alveolar cavities (Fig. S4), suggesting that this regimen is not appropriate for OMC.

Besides reducing ATCC 19977 lung burden, both the ERC and the OMC regimens had concomitant effects of reducing the extent of inflammatory lung injury, and notably, both ERC regimens could be effectively maintained to the 2-week endpoint (Fig. 6B through D).

### Change of MIC value after the end of the experiment

At the end of the experiment, we collected all of the strains for MIC detection, and the MIC values had no change from those values at the start of the experiment.

## DISCUSSION

As isolates of MAB have shown a high resistance rate to anti-TB drugs, attention has focused on the repurposed use of existing antibiotics. TGC, a tetracycline antibiotic, is one of the currently recommended antibiotics for treating MAB infection (38, 39). Although it was reported that the condition of 60% of patients improved after administering TGC, adverse reactions such as nausea and vomiting were observed in 94% of the cases (40).

Over the last few years, *in vitro* studies of ERC (a tetracycline-like TGC) have shown low MIC values against MAB (29, 41, 42), highlighting its promising effect against this bacterium. In addition, recent case reports and clinical observation studies have reported good clinical outcomes using ERC-containing regimens for the treatment of MAB lung disease (43, 44). However, these studies primarily provided MIC data (29, 41, 42) and did not systematically evaluate the pharmacodynamic properties, optimal dosing, or *in vivo* efficacy of ERC against MAB. More evidence from controlled preclinical models is needed to confirm the potential of ERC as a monotherapy or combination agent and to inform the design of clinical trials.

In this study, we conducted a comprehensive evaluation of the antimicrobial activity of ERC against MAB through *in vitro*, intracellular, and *in vivo* models. ERC demonstrated potent antibacterial activity against both reference and clinical strains of MAB *in vitro*, with MIC_50_ and MIC_90_ values consistent with those reports in other regions (29, 45). Our intracellular infection model revealed that ERC is effective not only against the reference strain but also against clinical isolates with pre-existing resistance to CLA or even TGC. The ability of ERC to inhibit a TGC-resistant isolate (KLS1YXN) is particularly promising, suggesting a potential role for ERC in the treatment of complex, multidrug-resistant MAB infections where current therapeutic options are severely limited. Time-kill and growth curve analyses further showed that increasing the concentration of ERC enhanced both the degree and duration of bacterial suppression. It is worth noting that the instability of tetracycline derivatives *in vitro* (46–48) may imply that the actual antibacterial activity of ERC could be even greater than measured. Obviously, these results strongly support ERC as a promising candidate for treating MAB infection.

Given the potent *in vitro* and intracellular activity of ERC, we further investigated its PAE. Remarkably, ERC exhibited prolonged, concentration-dependent PAE *in vitro*, lasting up to 10 days after a 2-hour pulse exposure to 16 µg/mL. This effect was longer than those induced by OMC and TGC at comparable concentrations. The subsequent observation of a substantial PA-SME further indicated that bacterial growth remains suppressed even at sub-inhibitory concentrations following prior exposure to a high dose of ERC. These *in vitro* findings were corroborated by *ex vivo* experiments in macrophages, where we observed a prolonged intracellular antibacterial activity after drug removal. Specifically, the inhibitory effect of ERC and OMC persisted for up to 1 week after drug washout, whereas the effect of TGC was short lived, with bacterial counts rebounding rapidly. This sustained intracellular activity, combined with the extended PAE and PA-SME observed *in vitro*, strongly suggests that ERC could support longer dosing intervals in clinical regimens. Such a strategy would allow more time for drug clearance between doses, potentially reducing the risk of drug accumulation and associated toxicities.

ERC is administered intravenously, which could pose challenges for long-term treatment regimens. Inspired by the work of Gumbo et al. (49), we evaluated a once-weekly high-dose regimen of ERC in a mouse model of MAB pulmonary infection. Notably, the weekly dosing regimen was as effective as the alternate-day regimen in reducing lung bacterial burden over 2 weeks. Importantly, the weekly regimen was more effective at inhibiting bacterial growth during the first week of treatment and was associated with reduced lung inflammation. No treatment-related mortality was observed in either the ERC group, whereas the OMC weekly dosing and TGC groups experienced mortality. Moreover, achieving high peak concentration with intermittent dosing may maximize bactericidal efficacy and reduce the potential for resistance development.

In this study, we observed a spontaneous decline in lung CFU counts in untreated animals. The experiment was concluded within 2 weeks, while pathological analysis confirmed that control mice remained infected, allowing for a valid comparison of treatment effects across groups.

The present study has some limitations. We did not use independent clinical MAB isolates to establish infection in mice, and it is recognized that different subspecies of MAB (e.g., *M. abscessus subsp. abscessus*, *M. abscessus subsp. massiliense*, and *M. abscessus subsp. bolletii*) may differ in virulence and drug susceptibility.

In conclusion, this study, through a multi-dimensional experimental design, has systematically demonstrated the potent antibacterial activity of ERC against MAB and its unique, long-lasting pharmacological properties. The combination of low MICs, strong intracellular activity, prolonged PAE, and the efficacy of a once-weekly dosing regimen in mice positions ERC as a highly promising candidate for the treatment of MAB infections. These findings not only offer a new therapeutic option for managing antibiotic-resistant MAB diseases but also provide a strong scientific rationale for exploring extended-interval dosing strategies in future clinical trials. The successful translation of these findings could significantly improve patient outcomes and reduce the public health burden associated with this difficult-to-treat pathogen.

## Data Availability

Upon a reasonable request, the raw data for the results presented in the manuscript are available from the corresponding author.

## SUPPLEMENTAL MATERIAL

Supplemental figures and tables spectrum.01473-25-s0001.pdf Figures S1 to S4 and Tables S1 to S3.

ASM does not own the copyrights to Supplemental Material that may be linked to, or accessed through, an article. The authors have granted ASM a non-exclusive, world-wide license to publish the Supplemental Material files. Please contact the corresponding author directly for reuse.
