# Supplementary material for: Eravacycline for Mycobacterium abscessus infections: pharmacodynamic advantages of long-acting post-antibiotic effects and weekly dosing regimens
Source: Microbiol Spectr. 2026 Mar 30;14(5):e01473-25. doi: 10.1128/spectrum.01473-25 (PMC13141830; doi:10.1128/spectrum.01473-25)
Supplement: Supplemental figures and tables — Figures S1 to S4 and Tables S1 to S3. [file spectrum.01473-25-s0001.pdf]

Table S1 Intracellular bactericidal activities of ERC, OMC and TGC at different concentrations

	NC	ERC10 μg/mL	ERC5 μg/mL	ERC1 μg/mL	OMC5 μg/mL	TGC5 μg/mL
M. abscessus ATCC 19977	6.992554	3.451545	4.60206	4.662347	4.845098	5.30103
	5.792392	3.30103	4.079181	4.643453	4.792392	5.350248
	5.869232	4.342423	4.30103	4.681241	5.292256	4.982271
	5.827369	3.60206	4	4.531479	4.819544	5.100371
mean survival (log_{10}CFU/mL)	6.120387	3.674264	4.245568	4.62963	4.937322	5.18348
**percent inhibition (%)**		**35.69948**	**29.21141**	**20.77203**	**14.77958**	**11.75655**
KL10TXF	4.623249	3.342423	3.414973	3.447158	3.924279	4.434569
	4.80618	3.146128	3.50515	3.991226	4.071882	4.531479
	4.778151	3.845098	3.869232	4.240549	4.25042	4.623249
	4.643453	3.892095	3.973128	4.025306	4.292256	4.485721
mean survival (log_{10}CFU/mL)	4.712758	3.556436	3.690621	3.92606	4.134709	4.518755
**percent inhibition (%)**		**24.536**	**21.68873**	**16.69295**	**12.26561**	**4.116557**
KLS1YXN	6.522966	4.748188	5.164353	5.633468	5.981366	6.556303
	6.46449	4.991226	5.041393	5.372912	5.993877	6.60206
	6.488269	4.944483	5.318063	5.480007	5.976808	6.531479
	6.46746	4.80618	5.033424	5.39794	5.902003	6.579784
mean survival (log_{10}CFU/mL)	6.485796	4.872519	5.139308	5.471082	5.963513	6.567406
**percent inhibition (%)**		**24.874**	**20.76056**	**15.64518**	**8.052714**	**-1.25829**
KL8HFZ	5.13226	2.60206	2.90309	3	3.30103	4.767898
	4.716003	2.778151	2.778151	3.30103	2.90309	3.662758
	4.823474	2.30103	2.90309	3.255273	3.146128	4.606381
	4.884229	3.20412	2.60206	3.447158	3.447158	5.579784
mean survival (log_{10}CFU/mL)	4.888992	2.72134	2.796598	3.250865	3.199352	4.654205
**percent inhibition (%)**		**44.3374**	**42.79807**	**33.50643**	**34.5601**	**4.802358**

Table S2 Different regimens for the treatment of bacterial CFUs in the lungs of mice infected with *M. abscessus* ATCC19977

weeks			NC			mean (log_{10}CFU/mL)
-1	6.112605	5.922206	5.795185	3.60206	3.30103	4.946617171
0	5.346353	5.158362	5.998259	5.647383	4.845098	5.399091
1	4.045323	3.176091	3.857332	4.697229	3.113943	3.7779836
2	2	3.230449	3.778151	4.30103	4.60206	3.582338032
W0-W1						1.6211074
W0-W2						1.816752968

ERC15mg/kg/day

-1	6.112605	5.922206	5.795185	3.60206	3.30103	4.946617171
0	5.346353	5.158362	5.998259	5.647383	4.845098	5.399091
1	2.954243	3.39794	3.146128	3.414973	2	2.98265678
2	1	1	3.30103	1	2	1.660205999
W0-W1						2.41643422
W0-W2						3.738885001
			ERC60mg/kg/week			
-1	6.112605	5.922206	5.795185	3.60206	3.30103	4.946617171
0	5.346353	5.158362	5.998259	5.647383	4.845098	5.399091
1	2	2	3.431364	3.361728	2	2.55861832
2	3.30103	2	2	2	1	2.060205999
W0-W1						2.84047268
W0-W2						3.338885001
			OMC15mg/kg/day			
-1	6.112605	5.922206	5.795185	3.60206	3.30103	4.946617171
0	5.346353	5.158362	5.998259	5.647383	4.845098	5.399091
1	2	2.954243	3.041393	3.278754	3.380211	2.930920007
2	1	1	2.477121	1	4.30103	1.95563025
W0-W1						2.468170993
W0-W2						3.44346075
			OMC60mg/kg/week			
-1	6.112605	5.922206	5.795185	3.60206	3.30103	4.946617171
0	5.346353	5.158362	5.998259	5.647383	4.845098	5.399091
1	3.838849	3.414973	3.591065			3.614962349
2	3.60206	2.477121	1	1	1	1.815836249
W0-W1						1.784128651
W0-W2						3.583254751
			TGC15mg/kg/day			
-1	6.112605	5.922206	5.795185	3.60206	3.30103	4.946617171
0	5.346353	5.158362	5.998259	5.647383	4.845098	5.399091
1	4.477121	3.70757	3.477121	3.778151	2.477121	3.583417038
2	1	1	2	1	1	1.2
W0-W1						1.815673962
W0-W2						4.199091

The integer 1 represented no colony growth on the plate and 2 represented 1 colony growth on the plate.

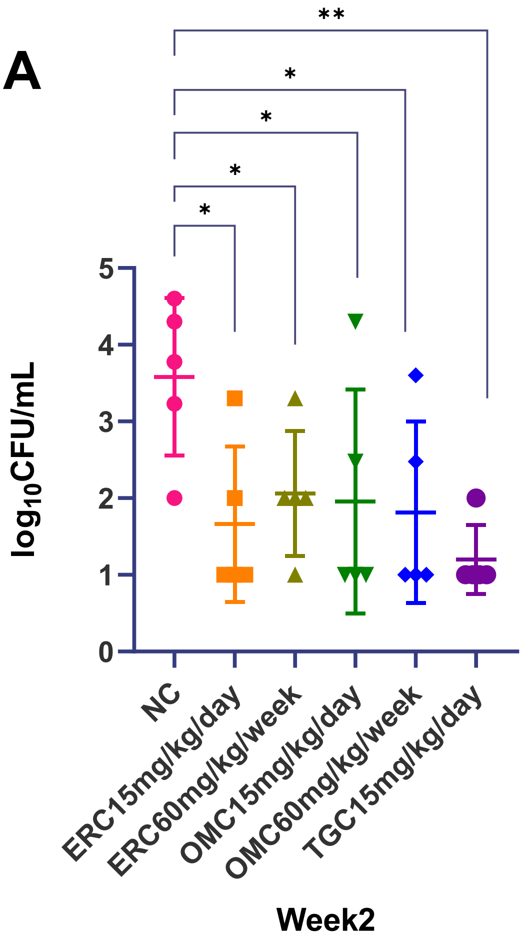

FIG S1 Intrapulmonary CFU counts in each group at the endpoint of the experiment.

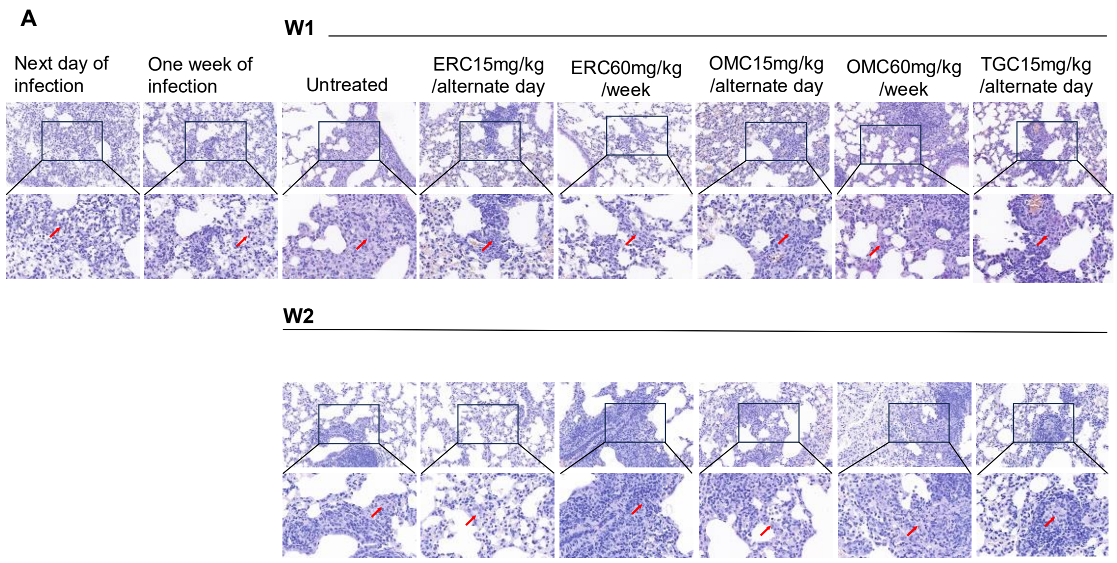

FIG S2 Acid-fast staining (A; scale bars, 20 mm[top] and 10 mm[bottom]) in the lungs of mice

infected with *M. abscessus* ATCC19977. The red arrow represents the bacillus that has been dyed red.

A

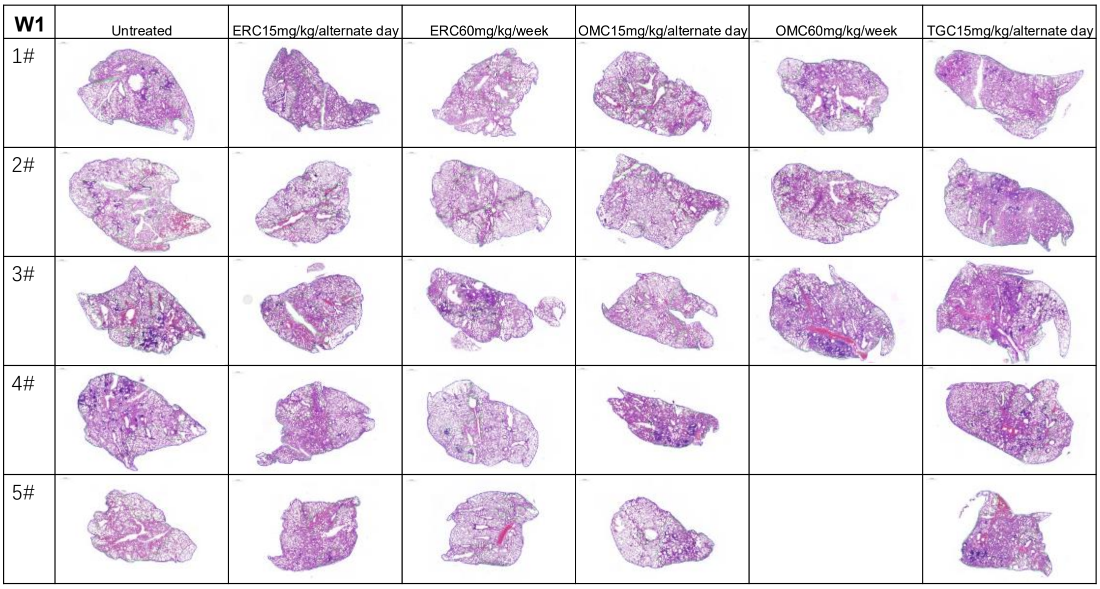

B

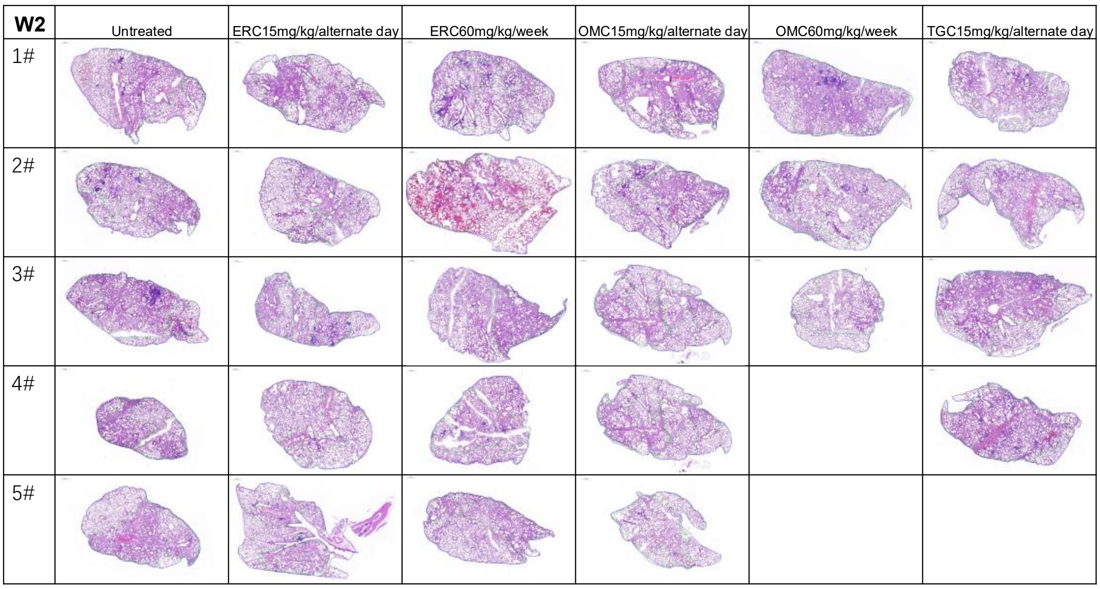

FIG S3 The solid blue line is the total area of the lung lobes, and the solid green line is the area of inflammation. The values measured using CaseViewer software can be found in Table S3.

Table S3 Quantitation of inflammatory damage areas.

W1	W2

	samples	Lung lobe area (μm²)	Inflammatory damage area (μm²)	Percentage inflammation (%)	samples	Lung lobe area (μm²)	Inflammatory damage area (μm²)	Percentage inflammation (%)
Untreated	1	18.5	10.51	56.81081081	1	23.4	14.86	63.5042735
	2	25.7	11.7	45.52529183	2	19.1	11.7	61.2565445
	3	17.1	10.14	59.29824561	3	20.4	13.57	66.51960784
	4	20.7	15.6	75.36231884	4	12.6	8.7	69.04761905
	5	15.2	9.7	63.81578947	5	19.3	12.8	66.32124352
ERC15mg/kg/alternate day	1	15.9	7.4	46.5408805	1	21	8.2	39.04761905
	2	15.9	3.58	22.51572327	2	21	8.9	42.38095238
	3	16.9	6.29	37.21893491	3	15	8	53.33333333
	4	17	6.59	38.76470588	4	21.3	5.5	25.82159624
	5	14.7	3.2	21.76870748	5	22.6	9.08	40.17699115
ERC60mg/kg/week	1	15.5	4.22	27.22580645	1	23.3	9.2	39.48497854
	2	19.6	3.17	16.17346939	2	32.1	17.8	55.4517134
	3	14.3	3.43	23.98601399	3	22.1	6.9	31.22171946
	4	20.6	2.46	11.94174757	4	19.8	10.7	54.04040404
	5	15.4	2.01	13.05194805	5	18.3	7.6	41.53005464
OMC15mg/kg/alternate day	1	18.2	5.85	32.14285714	1	21	15.4	73.33333333
	2	20	5.68	28.4	2	21.7	10.8	49.76958525
	3	15.1	5.03	33.31125828	3	22.7	11.9	52.42290749
	4	10.4	5.6	53.84615385	4	25.3	10.2	40.31620553
	5	13.7	5.12	37.37226277	5	15	9.2	61.33333333
OMC60mg/kg/week	1	17.1	13.3	77.77777778	1	24.8	20.1	81.0483871
	2	19.6	11.1	56.63265306	2	26.8	18.6	69.40298507
	3	23.7	17.9	75.52742616	3	16.9	11.7	69.23076923
TGC15mg/kg/alternate day	1	19.1	15.3	80.10471204	1	19.4	15.3	78.86597938
	2	21.5	16.5	76.74418605	2	19.5	12.87	66
	3	23.8	17.4	73.1092437	3	26.6	18.5	69.54887218
	4	21.8	9.9	45.41284404	4	21.3	17.8	83.56807512
	5	14.2	10.9	76.76056338				

A

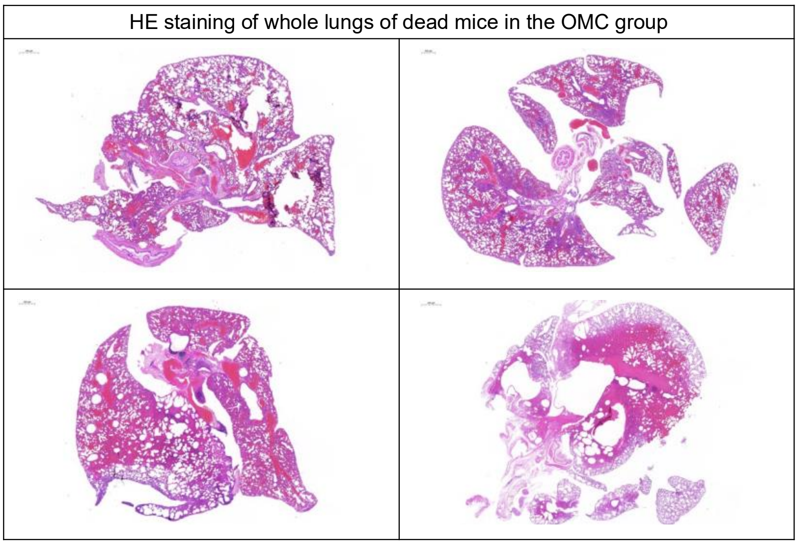

FIG S4 HE results showed massive congestion and haemorrhage in the lungs, with blood pooling in the alveolar cavities.
